# The Mouth Tells the Tale: Kaposi Sarcoma as the First Clue to Undiagnosed AIDS

**DOI:** 10.7759/cureus.85243

**Published:** 2025-06-02

**Authors:** Zaraq R Khan, Jacob Jarzynski, Urooj Nasim, Imad Majeed, Riya Shrestha

**Affiliations:** 1 Infectious Diseases, University of Louisville Hospital, Louisville, USA; 2 Internal Medicine, University of Louisville Hospital, Louisville, USA; 3 Research, University of Louisville Hospital, Louisville, USA

**Keywords:** hiv aids, immune reconstitution syndrome, immunosuppression, maxillofacial imaging, oral kaposi sarcoma

## Abstract

Kaposi sarcoma (KS) is a vascular neoplasm associated with human herpesvirus-8 (HHV-8), most commonly seen in individuals with advanced human immunodeficiency virus (HIV)/AIDS. We report the case of a 52-year-old male patient with no known HIV history who presented with a three- to four-month history of worsening sore throat, oral pain, diarrhea, weight loss, and systemic symptoms including night sweats and fatigue. The patient was eventually diagnosed with oral KS based on pathological results. This case highlights KS as a potential initial manifestation of advanced HIV/AIDS and the importance of early recognition and treatment.

## Introduction

Initially described in 1872, Kaposi sarcoma (KS) is an uncommon vascular neoplasm presumed to originate from endothelial cells, although some evidence suggests a possible lymphatic origin [1]. KS is associated with several conditions, including human herpesvirus 8 (HHV-8)-related viral oncogenes, cytokine-induced growth, and immunosuppression. Hallmarks of this sarcoma include neoangiogenesis, spindle cell proliferation, inflammation, and edema [2]. KS is the most prevalent human immunodeficiency virus (HIV)-associated malignancy [3].

KS is classified into four clinical variants: classic, African endemic, immunosuppression-associated (including transplant-associated), and AIDS-associated. The AIDS-associated variant represents the most aggressive form of the disease, predominantly affecting individuals infected with HIV type 1 (HIV-1), particularly among men who are homosexual and bisexual [4].

Clinically, KS lesions range from slow-growing to rapidly progressive. They may develop in multiple sites, including the skin (commonly the face and lower extremities), oral cavity, lymphatic system, and internal organs, most notably the lungs and gastrointestinal (GI) tract. The lesions are often discolored in hues of pink, red, or purple and can vary in size from a few millimeters to several centimeters. Morphologically, they may appear as flat macules, raised papules, nodules, or thickened plaques [5]. In the oral cavity, the palate, the gingiva, and the tongue are the most commonly affected sites [2, 4, 6]. Notably, oral lesions may serve as the first sign of KS in approximately 20% to 25% of cases and can be the sole manifestation or the earliest indicator of HIV infection [1].

## Case presentation

A 52-year-old White male patient with a past medical history of attention deficit hyperactivity disorder (ADHD), transaminitis, and seasonal allergies presented to the emergency department (ED) with a sore throat that had persisted for three to four months and had acutely worsened in the past month. His review of systems was notable for loss of appetite, oral pain with swallowing, nausea, and non-bloody diarrhea occurring three to four times per day, including episodes of incontinence. He also endorsed generalized weakness, fatigue, night sweats, and an unintentional weight loss of approximately 30 pounds over the preceding two to three months.

The patient’s social history was significant for chronic chewing tobacco use. He reported a distant history of unprotected heterosexual intercourse with partners of unknown HIV status and a history of fully treated syphilis 35 years prior. He denied intravenous drug use or recent travel.

On initial examination in the ED, the patient was ill-appearing and had a nonproductive cough. Oral examination revealed patchy white lesions on the tongue and buccal mucosa, a yellow-white eschar on the right hard palate, and two violaceous lesions on the left and right hard palate. Additionally, multiple scattered bleeding lesions and areas of scarring were noted on his extremities.

**Figure 1:**
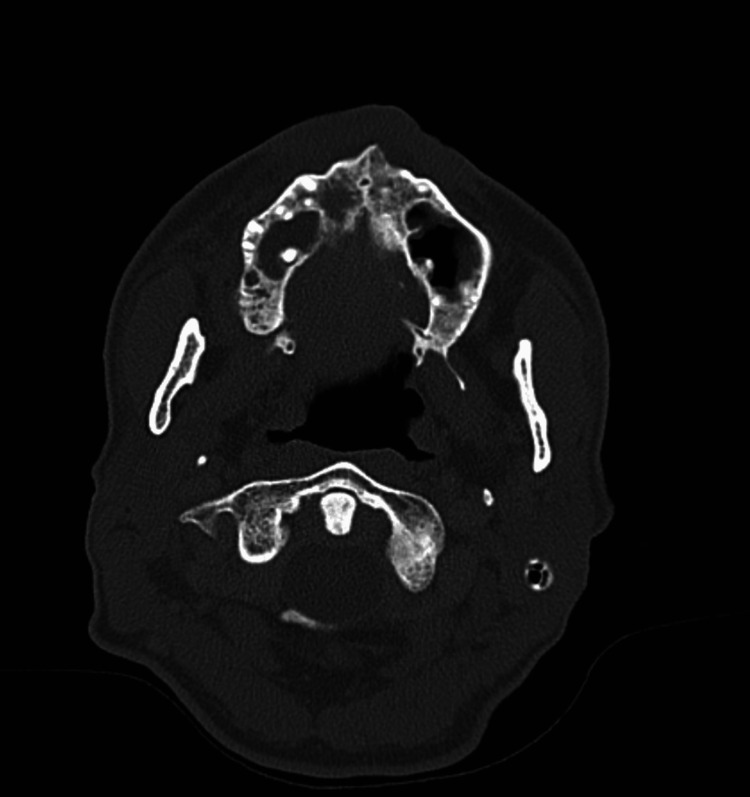
Erosion of the anterior aspect of the hard palate on the right side.

Initial laboratory findings were significant for a reactive HIV antibody screen, leukopenia (white blood cell (WBC) count of 4.3 × 10^9/L), and an elevated C-reactive protein (CRP) of 64 mg/L (Table 1). A maxillofacial CT scan obtained to further evaluate the oral lesions revealed erosion of the anterior hard palate, raising concern for osteomyelitis or an aggressive neoplastic process (Figure 1).

**Table 1:** The patient's key blood-based laboratory findings during hospitalization

Laboratory value	Patient level	Reference range
White blood cell count	4,300 cells/µL	4,500–11,000 cells/µL
Hemoglobin	11.5 g/dL	13.5–17.2 g/dL
Platelets	172,000 cells/µL	150,000–450,000 cells/µL
Creatinine	1.10 mg/dL	0.7–1.3 mg/dL
C-reactive protein (CRP)	64 mg/L	<10 mg/L
HIV RNA viral load	236,000 copies/mL	Undetectable
CD4 count (absolute and %)	3 cells/µL (1.3%)	500–1500 cells/µL

Though initially afebrile, the patient developed a fever of 103.1°F within 12 hours of ED presentation and was subsequently admitted for management of sepsis, diarrhea, and further workup in the context of newly diagnosed HIV. Empiric broad-spectrum antibiotics, vancomycin 1 gm every 12 hours and piperacillin-tazobactam 3.375g every eight hours, were initiated following collection of blood cultures and biopsy specimens. Vancomycin was discontinued on hospital day 2 after negative methicillin-resistant *Staphylococcus aureus* (MRSA) nasal screening.

During his seven-day hospitalization, the patient experienced persistent high fevers (Tmax 103.1°F), ongoing diarrhea (three to four loose stools/day), and night sweats. A comprehensive infectious disease workup-including blood cultures (aerobic, anaerobic, fungal, acid-fast bacilli (AFB)), serum cryptococcal antigen, oral lesion biopsy with tissue cultures (aerobic, anaerobic, fungal, AFB), sputum cultures, hepatitis serologies (hepatitis B surface antigen, hepatitis B surface antibodies, hepatitis B core antibodies, hepatitis A IgG antibodies and hepatitis C IgG antibodies), urine *Chlamydia*/*Gonorrhoea *polymerase chain reaction (PCR) panel, syphilis serologies (*Treponema pallidum* antibody and reactive plasma reagin), respiratory pathogen PCR, GI pathogen PCR, ova and parasite (O&P) stool studies, microsporidia exam, *Clostridioides difficile* toxin assay, *Cyclospora *and AFB stool smears, *Toxoplasma *serologies, and QuantiFERON-TB Gold-was unrevealing.

Despite broad-spectrum antibiotics, the patient's symptoms were refractory, and both blood and biopsy cultures remained negative. On hospital day 5, CD4 testing revealed profound immunosuppression (CD4 count = 3 cells/µL) and a viral load of 236,000 copies/mL. After multidisciplinary discussion, the patient was initiated on trimethoprim-sulfamethoxazole 800/160mg for prophylaxis against *Pneumocystis jirovecii* pneumonia (PJP) and *Toxoplasma gondii* and on bictegravir/emtricitabine/tenofovir alafenamide (Biktarvy) for antiretroviral therapy (ART), despite pending HIV genotyping. 

**Figure 2:**
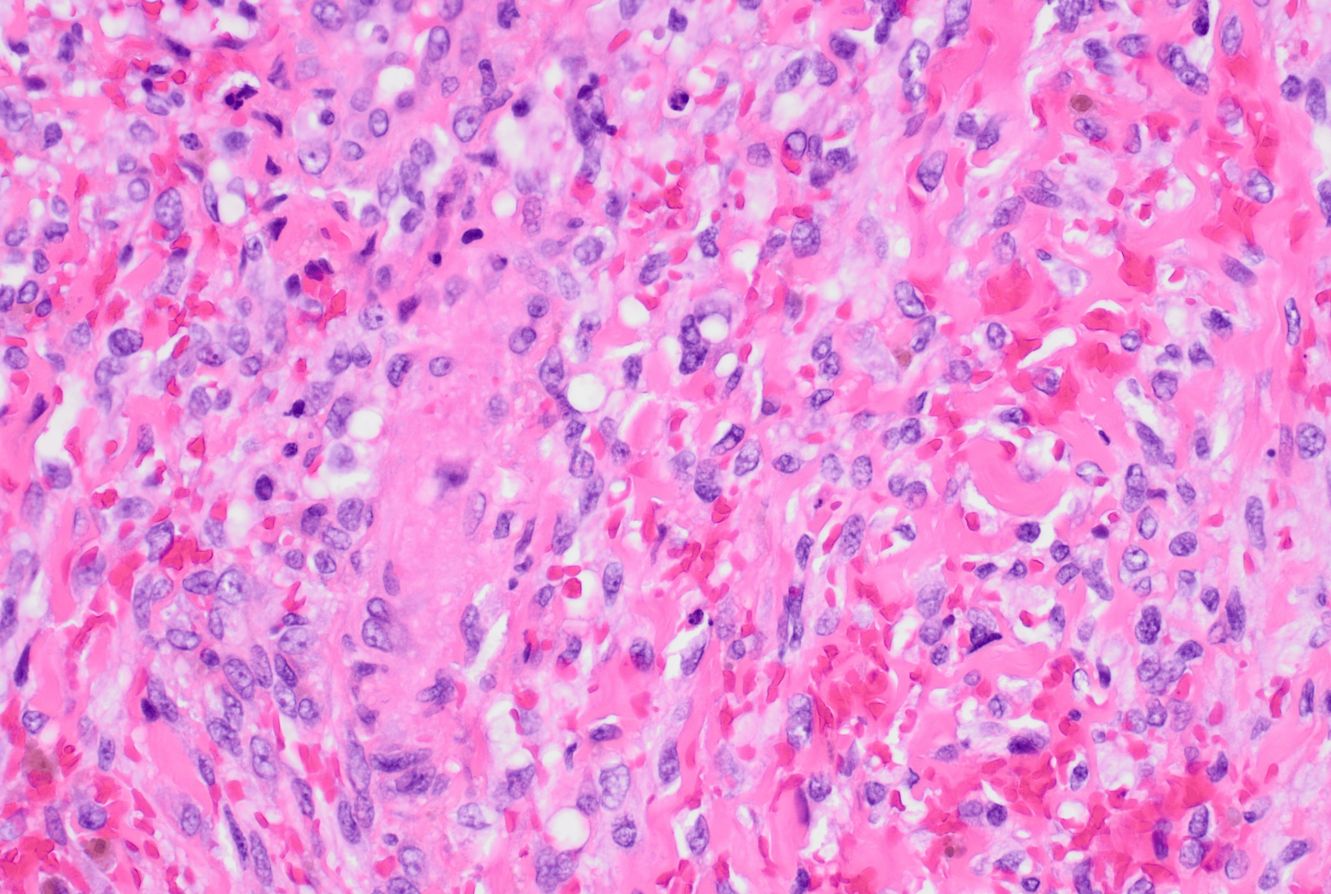
Positive immunohistochemical staining for CD31 of the spindle cells implies endothelial origin.

On hospital day 6, the immunostaining results from biopsy revealed submucosal spindle cells that were negative for CAM 5.2 (low molecular weight cytokeratin), 34 betaE12 (high molecular weight cytokeratin), p-40 (squamous cell carcinoma marker), and SOX-10 (melanoma marker), while they were positive for CD-31 (endothelial/vascular marker), supporting the morphological diagnosis of KS. The histopathology slides were also sent to the Joint Pathology Center (JPC) for HHV-8 staining, which came back positive in addition to D2-40 and ERG, thus reaffirming the diagnosis of KS (Figure 2).

With AIDS identified as the underlying cause of his symptoms and with ART and supportive care initiated, the patient was discharged on hospital day 7 in stable condition. He was referred for outpatient follow-up with Infectious Diseases for ongoing HIV/AIDS management and with Oncology for treatment of KS.

## Discussion

KS is an angioproliferative malignancy caused by HHV-8, with serologic confirmation of HHV-8 infection in approximately 95% to 98% of patients [7]. There are four epidemiologic forms of KS: AIDS-related (epidemic), African (endemic), organ transplant-associated, and classic. Of the four, oral lesions are most commonly seen in AIDS-related KS [8,9]. Additionally, oral lesions may also be the first clinical manifestation of undiagnosed HIV infection [8,9]. Oral KS is seen in approximately 22% of all individuals with KS [8,9]. Lesions are most often discovered on the hard palate (50%), followed by the gingiva (28%), and can be represented by both small, macular lesions or larger, infiltrative nodular lesions in some cases [9]. Although early oral lesions in KS are typically asymptomatic, more advanced disease can be painful, become at risk for infection, cause difficulty with speech and mastication, cause disfigurement, or cause dysphagia [10]. The violaceous macules of the hard palate appreciated on physical exam were consistent with the typical external features of oral KS. Additionally, his constellation of symptoms on presentation, including diarrhea, weight loss, and dysphagia, raised concern for advanced disease.

Albeit a presumptive diagnosis of KS can be made based on the observation of typical clinical features, the definitive diagnosis is made via tissue biopsy. This is an especially vital step to take since even typical clinical features can be easily confused with other conditions, including bacillary angiomatosis, hemangioma, pyogenic granuloma, inflammatory gingival enlargement, drug-induced gingival hyperplasia, and certain malignancies [10]. The histological characteristic of early macular KS consists of many abnormal small blood vessels dissecting tissues, while a biopsy of the later plaque stage would reveal proliferative spindle cells as well as further proliferation of blood vessels [10]. As one would expect, immunohistochemical stains of KS lesional cells are positive for endothelial markers such as factor VIII-related antigen, CD31, and CD34 [10]. The pathology report for the biopsy specimens of the hard palate of our patient, provided by the Joint Task Force National Capital Region Medical JPC, revealed proliferation of irregular, slit-like vascular channels and uniform spindle cells, with extensive extravasation of red blood cells, as well as hemosiderin deposition. On immunohistochemistry, cells were diffusely positive for CD31, D2-40, ERG, and HHV-8.

There is a staging system specifically for AIDS-related KS that consists of three categories: Tumor, Immune System, and Systemic Illness (TIS), scored 0-1, with 1 being considered a poorer risk [11]. The extent of oral involvement in KS is considered in the tumor category, with extensive oral involvement sufficient for one point. Other criteria that can be scored in this category include tumor-associated edema/ulceration, GI KS, and KS involvement in other non-nodal viscera. Immune system grading is based on CD4 count and is associated with poorer risk with a CD4 count <200/µL (150/µL has previously been suggested as having better discrimination for prognosis [12]. The last category, Systemic Illness, is scored based upon the presence of features such as B-type symptoms, thrush, or history of opportunistic infections. This scoring system was designed to indicate prognosis, with each additional scored category implying a worse prognosis. Previous studies have attempted to demonstrate the clinical value of each category in determining prognosis with mixed results [12,13]. Furthermore, with the continually increasing effectiveness of highly active antiretroviral therapy (HAART) in the treatment of HIV, it has been proposed that immune system grading does not possess the same prognostic power as it once had in the past [14]. Nevertheless, scoring using this format allows the practitioner to have an in-depth, patient-centered discussion about their prognosis as well as future management. In this case, the patient presented with features consistent with a poorer risk in each category.

Although symptomatology can help guide treatment selection, the mainstay of treatment of oral lesions in AIDS-related KS is the initiation of HAART as well as the early addition of systemic chemotherapy [10]. HAART not only aids in the treatment of oral KS, but it also likely provides a decreased risk of acquiring KS in HIV patients on therapy [7]. This differs from the other forms of KS, where local therapy is typically first-line treatment. This difference is due to the difficulty in controlling KS growth in the AIDS variant compared to the other epidemiological variants. Typical chemotherapy agents used in advanced, progressive AIDS-KS include liposomal anthracyclines, such as daunorubicin or doxorubicin; paclitaxel and etoposide remain as alternative agents for patients unable to tolerate anthracycline therapy [8]. For patients with limited mucocutaneous disease, the use of intralesional vinblastine has shown promise in limited trials [7]. Despite being very radiosensitive, radiotherapy is noted as a first-line treatment due to the risk of severe mucositis that has at times been life-threatening [11]. Our patient was initiated on HAART with Biktarvy and was treated concomitantly for oral thrush with fluconazole. He was then counseled regarding the potential of developing immune reconstitution inflammatory syndrome (IRIS) and instructed to establish care with oncology as an outpatient for further management of his KS and to follow up with the Infectious Diseases clinic upon discharge.

## Conclusions

This case illustrates the importance of maintaining a high index of suspicion for HIV/AIDS in patients with chronic systemic symptoms, mucocutaneous lesions, and unexplained immunosuppression. KS may be the initial manifestation of AIDS, even in individuals with distant or undocumented risk factors. Early diagnosis and prompt initiation of ART remain critical for improved outcomes.
